# Consensus commentary and position of the Italian Society of Nephrology on KDIGO controversies conference on novel anemia therapies in chronic kidney disease

**DOI:** 10.1007/s40620-024-01937-4

**Published:** 2024-05-06

**Authors:** Francesco Locatelli, Lucia Del Vecchio, Ciro Esposito, Loreto Gesualdo, Giuseppe Grandaliano, Maura Ravera, on behalf of the Collaborative Study Group on the Conservative Treatment of CKD of the Italian Society of Nephrology, Roberto Minutolo

**Affiliations:** 1 Department of Nephrology and Dialysis, Alessandro Manzoni Hospital, Lecco, Italy; 2 Department of Nephrology and Dialysis, Sant’Anna Hospital, ASST Lariana, Como, Italy; 3 Nephrology and Dialysis Unit, IRCSS Maugeri, University of Pavia, Pavia, Italy; 4 Department of Internal Medicine and Medical Therapy, University of Pavia, Pavia, Italy; 5 Renal, Dialysis and Transplantation Unit, Department of Precision and Regenerative Medicine and Ionian Area (DIMEPRE-J), University of Bari, Bari, Italy; 6 Dipartimento di Medicina e Chirurgia Traslazionale, Università Cattolica del Sacro Cuore, Rome, Italy; 7 Dipartimento di Scienze Mediche e Chirurgiche, U.O.C. Nefrologia, Fondazione Policlinico Universitario A. Gemelli IRCCS, Rome, Italy; 8 Nephrology, Dialysis and Transplantation Unit, Policlinico San Martino, Genoa, Italy; 9 Division of Nephrology, Department of Advanced Medical and Surgical Sciences, University of Campania, Luigi Vanvitelli, Piazza Miraglia, 80138 Naples, Italy

**Keywords:** Anemia, Hypoxia-inducible factor prolyl hydroxylase inhibitors, KDIGO, Position paper, HIF-PHI

## Abstract

Hypoxia-inducible factor prolyl hydroxylase inhibitors (HIF-PHIs) are new drugs developed for the treatment of anemia associated with chronic kidney disease (CKD). This class of drugs stimulates endogenous erythropoietin production and, at the same time, improves iron absorption and mobilization of iron stores (less evident with daprodustat, vadadustat and enarodustat). Several studies have been published in the last few years showing that these agents are not inferior to standard therapy in correcting anemia associated with CKD. The efficacy of HIF-PHIs is coupled with a safety profile comparable to that of standard erythropoiesis stimulating agent (ESA) treatment. However, studies with HIF-PHIs were not long enough to definitively exclude the impact of new drugs on adverse events, such as cancer, death and possibly cardiovascular events, that usually occur after a long follow-up period. Kidney Disease: Improving Global Outcomes (KDIGO) recently reported the conclusions of the Controversies Conference on HIF-PHIs held in 2021. The goal of the present position paper endorsed by the Italian Society of Nephrology is to better adapt the conclusions of the latest KDIGO Conference on HIF-PHIs to the Italian context by reviewing the efficacy and safety of HIF-PHIs as well as their use in subpopulations of interest as emerged from more recent publications not discussed during the KDIGO Conference.

**Graphical abstract:**

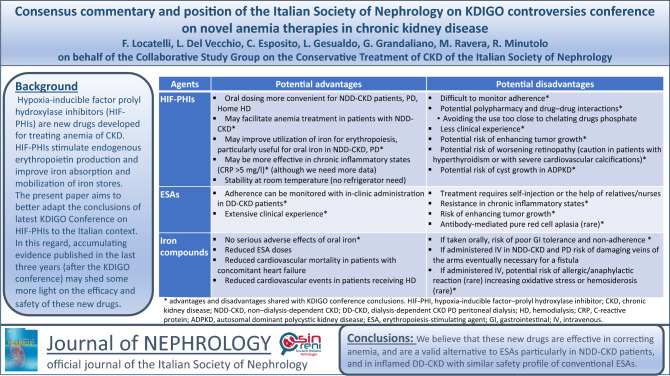

## Introduction

Guidelines are tools that should support physicians in making the best therapeutic decisions. Since 2003, the Kidney Disease: Improving Global Outcomes (KDIGO) has been setting standards and promoting the treatment of patients with chronic kidney disease (CKD).

However, the European perspective, often distinct, and frequently different across countries, is sometimes not adequately represented in the KDIGO Guidelines or Controversies Conferences, with a consequent need to adapt KDIGO recommendations in each country by acknowledging that a standardized approach may not be suitable in all cases [1].

With regard to anemia, in 2012, KDIGO published the guidelines for diagnosing and managing anemia in CKD [2]. The European Renal Best Practice (ERBP), a dedicated Committee of European Renal Associations, provided a Position Paper on these guidelines in 2013 [3]. Since then, no further KDIGO Guidelines or ERBP position statements on anemia have been released, even though an update of KDIGO guidelines is underway. Given the significant progress in anemia therapies, KDIGO organized two Controversies Conferences to review the latest evidence and its implications for managing anemia in clinical practice. The first conference in 2019 mainly focused on iron therapy, while the second one held in 2021, specifically discussed hypoxia-inducible factor-prolyl hydroxylase inhibitors (HIF-PHIs), a new class of agents for treating anemia [4, 5]. The present paper aims to better adapt the conclusions of the latest KDIGO Conference on HIF-PHIs to the Italian context. In this regard, accumulating evidence published in the last three years (following the KDIGO conference) may shed some more light on the efficacy and safety of these new drugs. In particular, new findings emerging from several meta-analyses [6–14], one trial in peritoneal dialysis patients [15] and some post-hoc and pooled analyses of randomized controlled trials (RCTs) [16–19] might be helpful for better placement of HIF-PHIs in the clinical practice.

## Efficacy of HIF-PHIs

### Opinion of the group

**As a group, we agree with the KDIGO Conference participants that HIF-PHIs are as effective as erythropoiesis-stimulating agents (ESAs) in correcting hemoglobin levels in non-dialysis dependent (NDD-CKD) and dialysis dependent (DD-CKD) populations.**

All ESAs effectively correct CKD-associated anemia by replacing erythropoietin (EPO) deficiency occurring in failing kidneys. Unlike ESAs, HIF-PHIs activate the hypoxia-inducible factor (HIF) pathway, thereby stimulating the transcription of the EPO gene and upregulating the expression of genes involved in erythropoiesis and in iron metabolism [20]. This peculiar mechanism of action was expected to correct anemia more effectively by increasing endogenous EPO production and simultaneously enhancing enteric iron absorption and iron mobilization (unlike ESAs). Furthermore, substantially lower peak serum EPO levels have been found in patients treated with HIF-PHIs in comparison with ESA treatment [21]. The majority of RCTs with HIF-PHIs demonstrated their non-inferiority as compared to ESAs. However, because of their study design, these RCTs cannot demonstrate superiority versus the standard of care [5]. In this regard, some recent meta-analyses (Table 1), not discussed by KDIGO Conference participants because they were published after the Conference, reported a larger increase of hemoglobin levels in patients receiving HIF-PHIs [6–11, 14], while hemoglobin target achievement did not differ [7, 12].

**Table 1:** Description of meta-analyses evaluating efficacy and safety of HIF-PHIs

Author (year)^ref^	Study type	Studies/ Patients	HIF-PHI	Control	Setting	Hb change (ΔHb) or Hb target achievement [95%CI]	Iron parameters (mean [95% CI])	Adverse outcomes (RR [95% CI])
Takkavatakarn (2023)^6^	Phase 2 Phase 3 Not published	46/29,855	Daprodustat Desidustat Enarodustat Molidustat Roxadustat Vadadustat	Placebo, ESA	NDD-CKD DD-CKD	Mean ΔHb (g/dL) vs control (placebo + ESAs) **0.66 [0.50–0.82]**	Hepcidin (ng/mL) **– 29 (– 34; – 24)** Iron (µg/dL) 2.2 (– 0.4;4.7) TIBC (µg/dL) **44 (39;49)** TSAT (%) **– 0.3 (– 3.7; – 2.3)** Ferritin (ng/mL) **– 43 (– 57; – 29)**	HIF-PHIs vs control (placebo + ESAs) MI 0.99 [0.87–1.14] Stroke: 0.97 [0.77–1.23] MACE: 1.00 [0.94–1.07] Thrombosis: **1.26 [1.00–1.57]** Hypertension: 0.99 [0.91–1.09] Hyperkalemia: 0.99 [0.88–1.11] ESKD: 1.06 [0.95–1.09] Death: 0.91 [0.78–1.07]
Minutolo (2023)^7^	Phase 3	26/24,387	Daprodustat Desidustat Enarodustat Molidustat Roxadustat Vadadustat	ESA	NDD-CKD DD-CKD	Mean ΔHb (g/dL) vs ESAs **0.10 [0.02–0.17]** Achievement of Hb target vs ESAs OR 1.04 [0.88–1.22]	Hepcidin (ng/mL) **– 19 (– 29; – 10)** Iron (µg/dL) **10 (6;14)** TIBC (µmol/L) **36 (32; 41)** TSAT (%) 0.3 (– 1.7; 1.0) Ferritin (ng/mL) **– 17 (– 32; – 1)**	Cancer: 0.93 [0.76–1.13] MACE: 1.00 [0.94–1.07] MACE + : 1.01 [0.95–1.06] Thrombosis: 1.08 [0.84–1.38] AVF thrombosis: 1.02 [0.93–1.13] Death: 1.02 [0.95–1.13]
Chen J (2023)^8^	Phase 2 Phase 3	26/15,020	Daprodustat Enarodustat Molidustat Roxadustat Vadadustat	Placebo, ESA	NDD-CKD DD-CKD	Mean ΔHb (g/dL) vs ESAs - Daprodustat – 0.14 [-0.45–0.16] - Enarodustat 0.24 [– 0.28 to 0.76] - Molidustat – 0.05 [– 0.45 to 0.34] - Roxadustat **0.32 [0.10–0.53]** - Vadadustat – 0.07 [– 0.43 to 0.29]	HIF-PHI vs ESA Daprodustat ↓ hepcidin TSAT and ferritin ↑ TIBC Enarodustat ↓ hepcidin ↑ TIBC Molidustat No change Roxadustat ↓ hepcidin and ferritin ↑ TIBC Vadadustat ↓ hepcidin ↑ TIBC	HIF-PHI vs ESA Hypertension - Vadadustat **0.74 [0.60–0.91]** - No difference for other HIF-PHIs Thrombosis - Daprodustat **2.52 [1.28–4.94]** - Roxadustat **1.61 [1.22–2.12]** - No difference for other HIF-PHIs
Chen H (2021)^9^	Phase 2 Phase 3	30/13,146	Daprodustat Desidustat Enarodustat Molidustat Roxadustat Vadadustat	Placebo, ESA	NDD-CKD DD-CKD	WMD Hb vs ESA **0.13 [0.03–0.22]**	Hepcidin (SMD) **– 12 (– 23; – 1)** Iron (SMD) **0.2 (0.1; 0.4)** TIBC (SMD) **0.7 (0.5; 1.0)** TSAT (WMD) 0.8 (– 0.02;1.5) Ferritin (SMD) **– 0.1 (– 0.2; – 0.0)**	HIF-PHIs vs ESA MACE: 1.02 [0.90–1.14] Heart failure: 0.95 [0.72–1.26] Hypertension: 1.02 [0.89–1.17] Hyperkalemia: 1.14 [0.69–1.89] Thrombosis: **1.31 [1.05–1.63]**
Zheng (2020)^10^	Phase 2 Phase 3	19/2,768	Daprodustat Desidustat Enarodustat Molidustat Roxadustat Vadadustat	Placebo, ESA	NDD-CKD	Mean ΔHb (g/dL) vs placebo - Desidustat **2.46 [0.93–3.99]** - Enarodustat **1.81 [0.87–2.75]** - Molidustat **1.68 [0.64–2.72]** - Roxadustat **1.61 [0.99–2.22]** - Daprodustat **1.55 [0.74–2.36]**	NA	HIF-PHI vs placebo Death: No difference
Chen D (2023)^11^	Phase 3	19/14,947	Daprodustat Desidustat Enarodustat Molidustat Roxadustat Vadadustat	ESA	DD-CKD	Mean ΔHb (g/dL) vs ESAs - Daprodustat 0.04 [– 0.11; 0.19] - Desidustat 0.14 [– 0.24; 0.52] - Enarodustat 0.06 [– 0.28; 0.40] - Molidustat – 0.30 [– 0.02; 0.61] - Roxadustat **0.19 [0.07; 0.30]** - Vadadustat – 0.11 [– 0.07; 0.30]	HIF-PHI vs ESA Roxadustat ↓ hepcidin Vadadustat ↓ TSAT	HIF-PHI vs ESA CV events: No difference Hypertension - Vadadustat **0.81 [0.68–0.97]** - No difference for other HIF-PHIs Hyperkalemia: No difference Cancer: No difference AVF complications - Roxadustat **1.15 [1.04–1.27]** - No difference for other HIF-PHIs Gastrointestinal disorders - Enarodustat **6.92 [1.51–31.69]** - No difference for other HIF-PHIs
Natale (2022)^12^	Phase 2 Phase 3	51/30,994	Daprodustat Desidustat Enarodustat Molidustat Roxadustat Vadadustat	Placebo, ESA	NDD-CKD DD-CKD	Achievement of Hb target vs ESAs RR 1.00 [0.93–1.07]	NA	HIF-PHIs vs ESA CV death: 1.05 [0.88–1.26] MI: 0.91 [0.73–1.10] Stroke: 1.06 [0.71–1.56] Thrombosis: 1.09 [0.86–1.39] Hyperkalemia: 0.92 [0.82–1.04] Cancer: 0.83 [0.43–1.59] Kidney failure: 1.02 [0.91–1.15] Death: 0.98 [0.91–1.06]
Zheng (2023)^13^	Phase 2 Phase 3	23/15,144	Daprodustat Desidustat Enarodustat Molidustat Roxadustat Vadadustat	Placebo, ESA	NDD-CKD DD-CKD	NA	NA	HIF-PHIs vs ESA Cardiac AEs: 1.06 [0.98–1.14] Hypertension: **0.89 [0.81–0.98]** Hyperkalemia: 0.92 [0.81–1.04] ESKD: 0.99 [0.91–1.08] Death: 1.01 [0.91–1.12]
Abdelazeem (2021)^14^	Phase 2 Phase 3	10/5,768	Roxadustat	Placebo, ESA	DD-CKD	SMD Hb vs control (placebo + ESAs) **0.21 [0.02–0.39]**	Hepcidin (SMD) **– 16 (– 28;-3)** Iron (SMD) **0.3 (0.2; 0.4)** TIBC (SMD) **0.8 (0.6; 1.0)** TSAT (WMD) 0.04 (– 0.04; 0.11) Ferritin (SMD) – 0.1 (– 0.2; 0.1)	HIF-PHI vs control (placebo + ESA) CV adverse event: 1.03 [0.95–1.11] Hypertension: 1.04 [0.90–1.19] Hyperkalemia: 1.07 [0.85–1.35]

Bold indicates significant differences

*ESAs* erythropoiesis stimulating agents, *CKD* chronic kidney disease, *NDD-CKD* non-dialysis-dependent CKD, *DD-CKD* dialysis-dependent CKD, *NA* not assessed, *CV* cardiovascular, *MI* Myocardial infarction, *Cardiac AEs* cardiovascular death, MI, unstable angina, ischemic stroke, heart failure, arrhythmia, or other cardiac and valvular diseases reported in the articles, or major adverse cardiovascular events (MACE) or enlarged MACE (MACE +) or “cardiac disorders” reported in the included studies; *ESKD* end-stage kidney disease, *WMD* weighted mean difference, *SMD* standardized mean difference, *AVF* arteriovenous fistula

It is important to note that RCTs also differed with regard to iron supplementation strategies among RCTs and within the same RCT. Therefore, the amount of iron administered to patients and their iron stores may have varied between the HIF-PHI and ESA arms. These discrepancies, together with HIF-PHI doses administered, are important confounding factors affecting the reliability of comparison.

## Side effects of HIF-PHIs

### Opinion of the group

**As a group, we agree with the KDIGO Conference participants that the potential risks associated with the use of these drugs must be carefully balanced against their benefits in treating anemia in CKD patients. However, the statement in their conclusions** ***“In terms of cardiovascular safety, HIF-PHIs are inferior to, or at best similar to conventional ESAs”*** **is a matter of discussion, because, in our opinion, accumulating evidence in the two years after the KDIGO conference supports the non-inferiority of HIF-PHIs for cardiovascular safety. Other safety issues require close monitoring and further research to attain long-term safety data to better understand the overall impact of HIF-PHIs on patient outcomes.**

The pharmacological activation of the HIF pathway in patients with anemia of CKD has effects beyond erythropoiesis and iron metabolism. Indeed, depending on the properties of the administered drug, dosing, and exposure, HIF-PHIs can potentially affect various cellular processes, including cellular differentiation and growth, vascular homeostasis and hemodynamics, inflammation, and cellular metabolism [20]. The activation of non-erythropoietic signaling pathways by HIF-PHIs in patients is challenging to predict and measure. The following safety concerns regarding HIF-PHIs should be considered.

1. *Cardiovascular outcomes*. Overall, most RCTs have consistently demonstrated the non-inferiority of HIF-PHIs versus controls for major adverse cardiovascular events (MACE) in the NDD-CKD population. Different HIF-PHIs and study settings have yielded different results, and potential explanations for these diverse effects include imbalances in patient characteristics, geographic location and study design [19, 22]. Indeed, it is important to note that placebo-controlled studies (all with roxadustat) were characterized by a large and significant difference in discontinuation rate (59% in placebo arms versus 38% in roxadustat arms) leading to a marked disparity in duration of drug exposure and consequently to a higher number of events among roxadustat-treated patients [22]. In contrast, in the ESA-controlled trials involving the DD-CKD population, the general consensus was that HIF-PHIs met non-inferiority criteria for MACE in cardiovascular outcome trials, in both incident and prevalent dialysis patients. The meta-analyses available after the KDIGO Conference confirmed the lack of increased risk of MACE with HIF-PHIs [6, 7, 9, 11–14] (Table 1). Similarly, pooled analyses of roxadustat studies and vadadustat trials did not highlight any increased risk in MACE and mortality [16, 17].
2. *Thromboembolic events*. Concerns have been raised on the risk of thrombosis associated with HIF-PHIs. The underlying mechanisms for this adverse effect have yet to be entirely understood. They are possibly related to either a more rapid rise of hemoglobin induced by the selected starting dose of some HIF-PHIs or to an influence of these drugs on the HIF target genes modulating coagulation, fibrinolysis and thrombus resolution [23]. However, most RCTs did not report any imbalance of thromboembolic events between HIF-PHIs and standard of care. Only one study on DD-CKD patients comparing roxadustat with ESAs reported an increased incidence of arteriovenous fistula thrombosis and deep vein thrombosis in the roxadustat arm [24]. The meta-analyses reported conflicting results [6–9, 12] (Table 1). However, besides the starting doses of HIF-PHIs, the amount of iron used and the hemoglobin levels reached should be considered when interpreting these findings.
3. *Hypertension*. Randomized controlled trials and recent meta-analyses did not evidence an increased risk of hypertension with HIF-PHIs in comparison with ESAs [6, 8, 9, 11, 13, 14] (Table 1).
4. *Kidney disease progression*. No study has evaluated the impact of HIF-PHIs on kidney disease progression as the primary outcome. Secondary analyses of RCTs consistently reported no significant effects on renal outcomes [27, 28]. In an RCT comparing roxadustat with placebo, the eGFR declined faster (*P* = 0.046) in the roxadustat group (– 3.7 mL/min/year) as compared to placebo (– 3.2 mL/min/year), but the higher discontinuation rate in the placebo group may have strongly affected this result [29]. Meta-analyses did not report any significant effect of HIF-PHIs on renal outcomes [6, 12, 13]. However, it should be underlined that most RCTs in NDD-CKD patients enrolled a high percentage of stage 5 CKD patients, thus precluding the possibility of adequately testing CKD progression.
5. *Malignancy risk.* Only one trial reported an increased risk of cancer-related death or tumor progression or recurrence in patients receiving daprodustat versus darbepoetin (3.7% vs 2.5%), but this difference disappeared in post-hoc analysis accounting for different dosing frequency in the darbepoetin arm (3.7% vs 3.5%) [28]. Overall, there is no consistent signal across HIF-PHIs regarding an excess risk of malignancy [7, 11, 12] (Table 1).
6. *Infection*. An increased incidence of sepsis and septic shock has been reported with roxadustat in the pooled analysis performed by the Food and Drug Administration (FDA) which also included placebo-controlled trials (2.25 events/100 pt-years with roxadustat vs 0.95 events/100 pt-years with placebo) [30]. A similar finding was reported by the European Medicines Agency (EMA) analysis in comparison with placebo (1.3 events/100 pt-years in the roxadustat group vs 0.95 events/100 pt-years in the placebo group); however, when roxadustat was compared with ESAs, the incidence rate of sepsis did not differ (2.0 events/100 pt-years with roxadustat vs 1.8 events/100 pt-years with ESAs) [31].
7. *Worsening of diabetic retinopathy.* It has been postulated that a worsening of diabetic retinopathy and macular degeneration could occur based on the angiogenetic effect of HIF-PHIs mediated by increased transcription of vascular endothelial growth factor (VEGF) [32, 33]. Available RCTs did not report any ophthalmologic impact of HIF-PHIs [34–36], a finding also confirmed by the Cochrane meta-analysis [12].
8. *Other safety concerns*. Inconsistent results have been reported with HIF-PHIs on additional safety issues, such as hyperkalemia, central hypothyroidism, cyst enlargement in polycystic kidney disease, and excess copper.

## Meta-analyses on the efficacy and safety of HIF-PHIs

### Opinion of the group

**Several meta-analyses have been published after the KDIGO conference and these are useful to shed some light on the efficacy and safety of HIF-PHIs. These studies report that HIF-PHIs are superior to placebo and non-inferior to ESAs in increasing and maintaining hemoglobin levels among dialysis and non-dialysis patients. Although all the meta-analyses published to date are largely reassuring with regard to the safety profile of HIF-PHIs, we believe that post-marketing surveillance should be mandatory to confidently confirm the absence of clinically relevant risks.**

Both efficacy and safety of HIF-PHIs have been assessed in nine meta-analyses that uniformly confirmed that HIF-PHIs are effective in correcting anemia in the whole spectrum of CKD in the absence of clear signals on safety issues [6–14] (Table 1). None of these meta-analyses was discussed in the KDIGO paper because most of them were published after the conference was held (December 2021).

It is important to keep in mind that these meta-analyses were heterogeneous in terms of type of study selected, CKD setting, type of HIF-PHIs, design of analysis and outcome. Interestingly, the most recent meta-analysis including only phase 3 RCTs with an active control arm (ESAs) lasting more than 24 weeks [7] reported that the increase in hemoglobin level is only evident when compared to short-acting ESAs (+ 0.21 g/dL) and absent when compared to long-acting ESAs (– 0.01 g/dL) [7]. When considering the achievement of hemoglobin target as an efficacy outcome, no differences emerged between HIF-PHIs and ESAs (Table 1) [7, 12].

Six meta-analyses of HIF-PHI trials reported a significant reduction of hepcidin and ferritin levels and an increase of total iron binding capacity in patients receiving HIF-PHIs, consistent with their mechanism of action [6–9, 11, 14]; changes in serum iron and transferrin saturation were less predictable (Table 1). These effects translate into lower intravenous iron dosing with HIF-PHIs but the degree of this iron sparing effect is too small to be considered clinically meaningful [7]. Furthermore, the paucity of data on oral iron therapy strongly limits the evaluation of the clinical impact of HIF-PHIs on NDD-CKD. Indeed, in these patients, a trial of oral iron is recommended but frequently omitted, likely due to either the therapeutic inertia of the nephrologists or the gastrointestinal side effects [37–39]. Studies from clinical practice are mandatory to evaluate whether improving iron metabolism with these drugs can translate into a reduction of iron use (lower doses, shorter oral courses and fewer intravenous injections).

No significant increase in cardiovascular risk has been reported in published meta-analyses focusing on HIF-PHI safety compared to ESA [6, 7, 9, 12–14] (Table 1). The lack of risk is consistent when MACE and MACE + were assessed by either rate ratio or hazard ratio [7] and when the single components of MACE and MACE + were analyzed separately [12]. Similar reassuring data were also reported by others [6, 13].

Meta-analyses have provided conflicting results on thrombotic risk (Table 1). The reason for this discrepancy is not readily apparent and it could be related to differences in the definition of the outcome, the difference in the control group or in the dosing of HIF-PHIs. The risk of malignancy did not differ between HIF-PHIs and ESAs [7, 12] (Table 1), even though the follow-up of included trials was relatively short to confidently estimate cancer risk. Finally, mortality risk was not increased with HIF-PHIs [6, 7, 10, 12, 13]. However, the proper assessment of outcomes requiring longer follow-up to occur needs extensive post-marketing surveillance to help clinicians detect potential harms.

## Effect of HIF-PHIs on health-related quality of life (HRQoL)

### Opinion of the group

**HRQoL has been evaluated only in RCTs with roxadustat and daprodustat. We believe that the effect on vitality score was negligible for roxadustat and of modest size for daprodustat, despite a significant increase in hemoglobin levels compared to placebo. Available evidence does not support a meaningful difference in HRQoL between HIF-PHIs and ESAs likely because the patients in the two arms achieved similar hemoglobin levels. The oral administration route could be perceived as an advantage in HRQoL by some NDD-CKD, home dialysis, peritoneal dialysis patients and kidney transplant recipients, especially for those experiencing injection pain or needle phobia. Unfortunately, the effect of the route of administration is not captured by the HRQoL instruments.**

Several RCTs with roxadustat included the 36-item Short-Form Survey (SF-36) assessment as an exploratory or secondary endpoint [29, 40–43]. However, these studies differed with regard to scoring system, trial design (double-blind vs open label) and comparator (placebo or ESA), thus precluding correct assessment of HRQoL. In particular, compared to placebo, roxadustat did not provide a clinically meaningful improvement in SF-36 vitality and physical functioning sub-scores despite better anemia control [29, 40, 41]. The same occurred in studies comparing roxadustat to ESAs [42, 43]. At the time of the KDIGO Conference, data from the ASCEND-NHQ study were available only in abstract form, while the full paper became available in 2023 [44]. This was a multicenter, double-blind RCT of 614 ESA-naïve patients with NDD-CKD and anemia (hemoglobin 8.5–10.0 g/dL) randomized to daprodustat or placebo to achieve a target hemoglobin level of 11–12 g/dL. SF-36 Vitality score (fatigue) change was a key secondary endpoint. It increased by 7.3 points with daprodustat and by 1.9 points with placebo (*P* = 0.0005) with more patients showing an increase in the SF-36 score of ≥ 6 points in the daprodustat than in the placebo group (58% vs 40%). Overall, the effect was clinically significant but modest, and thus not necessarily perceived as truly important by the patient [45]. Moreover, these results do not provide information about how the patients’ activities were affected [45]. The study design does not clarify whether the HRQoL improvement was due to anemia correction or if it was drug-specific, and whether the effect would apply at lower hemoglobin targets (11–12 g/dl is the upper limit of the target suggested by the ERBP and above that recommended by KDIGO).

Finally, the availability of an oral drug to treat CKD-related anemia may represent a potential tool to improve patients’ quality of life, especially for those with needle-phobia and/or requiring support for ESA injection. A further advantage involving all patients traveling for leisure or work is the lack of need to maintain the cold chain for storing HIF-PHIs, unlike with ESAs.

## Differences among HIF-PHIs

### Opinion of the group

**We believe that HIF-PHIs are all effective in correcting and maintaining hemoglobin levels, but their pleiotropic effects and their safety profiles are possibly dependent on the molecule and, for this reason, they should be evaluated individually by evaluating risks and benefits. In the era of precision medicine, the differences between these molecules may allow the nephrologist to find the most suitable treatment for each patient, reducing the side effects and the potential risks. Unfortunately, such an approach is now unfeasible because regulatory agencies made discrepant decisions on approving these drugs across countries.**

Six types of HIF-PHIs completed phase 3 clinical trial programs and were approved in various countries for the treatment of CKD-related anemia (Table 2). Although they share the same mechanism of action, these drugs show differences that were only partly taken into account by the KDIGO conference [5].

**Table 2:** Differences in main pharmacologic profiles among HIF-PHIs with Phase 3 data

Compound	Half-Life (hours) healthy/CKD	Starting dose	Dosing Schedule	Activity on PHD isoforms	Countries with marketing authorization (setting)
Daprodustat	~ 1/7	1–4 mg (ND) 4–12 mg (DD)	QD	PHD3 > PHD1 > PHD2	Japan (NDD-CKD, DD-CKD) EU (DD-CKD) US (DD-CKD)
Desidustat	7–11.4/-	100 mg	TIW	–	India (NDD-CKD, DD-CKD)
Enarodustat	9.0–11.3/14.8–15.9	2 mg (ND,PD) 4 mg (HD)	QD	PHD1 > PHD2 > PHD3	Japan (NDD-CKD, DD-CKD)
Molidustat	4–10/-	75 mg	QD	PHD3 > PHD1/PHD2	Japan (NDD-CKD, DD)
Roxadustat	16.0/16.8–18.5	70 mg (if b.w. < 100 kg) 100 mg (if b.w. ≥ 100 kg)	TIW	PHD1 = PHD2 = PHD3	China (NDD-CKD, DD-CKD) Japan (NDD-CKD, DD-CKD) Chile (NDD-CKD, DD-CKD) EU (NDD-CKD, DD-CKD) South Africa (NDD-CKD, DD) Russia (NDD-CKD, DD-CKD) South Korea (NDD-CKD, DD-CKD) Turkey (NDD-CKD, DD-CKD) Middle East (NDD-CKD, DD-CKD)
Vadadustat	4.7/7.9–9.1	300 mg	QD	PHD3 > PHD1 > PHD2	Japan (NDD-CKD, DD) EU (DD)

*CKD* chronic kidney disease, *NDD-CKD* non-dialysis-dependent CKD, *DD-CKD* dialysis-dependent CKD, *b.w.* body weight, *QD* once daily, *TIW* thrice weekly, *PHD* prolyl hydroxylase domain

HIF-PHIs are reversible inhibitors of all three prolyl-hydroxylase domain (PHD) isoforms but their enzyme inhibitory effect varies, with roxadustat showing an effect on all three PHD isoforms (Table 2). The pharmacokinetics are also different, so the effective dosing schedules vary, with roxadustat and desidustat being administered thrice weekly, compared to once-daily administration for daprodustat, enarodustat, molidustat, and vadadustat (Table 2). The lack of data directly comparing two or more HIF-PHIs does not allow for drawing firm conclusions about differences in the efficacy and safety of these compounds. As correctly underlined in the KDIGO conference report [5], the large variability of baseline levels of hepcidin, serum iron, transferrin saturation and ferritin among individual trials makes it difficult to carry out a proper comparison of the effect of different HIF-PHIs on iron parameters.

HIF-PHIs may affect lipid metabolism by increasing the degradation of 3-hydroxy-3-methylglutaryl coenzyme A reductase (the same target of statin therapy), thus reducing cholesterol levels. This ancillary effect has been shown in several phase 3 RCTs (particularly with roxadustat). However, its magnitude is relatively small for LDL-cholesterol in both NDD and DD populations (– 11 mg/dL, 95% CI – 15 to – 6) [7]. However, the degree of LDL decline, albeit statistically significant, is not large enough to modify cardiovascular outcome, particularly in DD-CKD patients for whom the role of cholesterol is less important. In addition, the reported decline in HDL level further limits the potential impact on cardiovascular prognosis [7].

Some differences observed among HIF-PHIs are due to study design, duration and timing of the target assessment. Furthermore, not all molecules were compared with the same type of ESA and, in the same studies, different ESAs were used as comparators, with dosages not being pre-specified but rather modulated according to local practice. Finally, studies using daprodustat, roxadustat, and vadadustat enrolled about 90% of patients; this lack of balance may not allow a correct comparison between the molecules. The differences in anemia correction reported by trials using different HIF-PHIs were considered almost negative points for the whole class of drugs by the KDIGO controversies conference [5].

## Use of HIF-PHIs in subpopulations of interest

### Hyporesponsive patients with inflammation

#### Opinion of the group

**In contrast with KDIGO conference participants who felt that the available data was insufficient at that time to demonstrate an effect of HIF-PHIs in the hyporesponsive population, in our opinion, available data do support the hypothesis that HIF-PHIs are more effective than ESAs for correcting anemia in hyporesponsive patients due to inflammation.**

HIF-PHIs suppress hepcidin release and improve iron metabolism [4]. In this regard, it has recently been reported that hepcidin decrease was not associated with hemoglobin increase [7], suggesting a direct effect of HIF-PHIs on hepcidin. Therefore, due to their mechanism of action, HIF-PHIs may be a better choice for ESA-hyporesponsive subjects.

Initial phase 2 studies and placebo-controlled trials have suggested that the efficacy of HIF-PHIs in correcting anemia is unaffected by inflammatory status [21, 29, 40, 41]. This hypothesis was subsequently tested in secondary analyses of phase 3 active-controlled studies in NDD and DD patients, showing that hemoglobin increase induced by HIF-PHIs did not differ between patients with higher or normal C-reactive protein (CRP) levels [15, 24, 28, 36, 42, 46–54]. In addition, HIF-PHI doses required to maintain hemoglobin within the target range were not influenced by CRP levels, while ESA-treated patients with high CRP required increased ESA doses to maintain hemoglobin levels [47–49, 51]. Hemoglobin response to HIF-PHIs, regardless of CRP levels, has been described in phase 3 studies using either roxadustat, molidustat, daprodustat, enarodustat or vadadustat, thus suggesting a class rather than a drug-specific effect. Experimental studies clarified that a decreased production of hepcidin and other inflammatory cytokines are the mechanisms supporting the ability of HIF-PHIs to correct anemia during inflammation [55, 56].

It is important to note that no data are available for patients with very high CRP levels because patients with rheumatoid arthritis, inflammatory bowel disease or heart failure were excluded from trials with HIF-PHIs. The same occurred for patients with recent infectious episodes or severe cardiovascular disease. Nonetheless, we believe that data do not sufficiently support the sentence in the KDIGO report “CRP concentrations that were considered high in trial participants were only slightly elevated” [5]. Indeed, none of the studies stratifying patients by CRP level above or below the normal range reported any metrics (mean, median or range) in the subgroup with CRP above the upper normal limit [24, 28, 29, 40–42, 46–54]. The same occurred when patients were stratified according to quintiles of CRP [56, 57]. Conversely, such information would be essential for nephrologists to properly characterize this “inflamed population”, whether on dialysis or not, and to establish at which level of inflammation HIF-PHIs are expected to be effective.

### Hyporesponsive patients with iron deficiency

#### Opinion of the group

**We deem that a definitive interpretation of iron-related data is difficult because a great deal of variability exists in trial design and iron administration practices. Although potential benefits can be expected with HIF-PHIs on iron metabolism, we believe that the patients to be treated with HIF-PHIs should be iron replete and that HIF-PHI therapy will not eliminate the need for iron replacement, especially in dialysis patients, because the sparing effect of intravenous iron is statistically significant but not clinically meaningful. In NDD-CKD, the effect of HIF-PHIs in reducing the need for oral iron supplementation (in terms of frequency and dosage) needs to be better defined, although potentially true based on its mechanism of action. The possibility of reducing doses or frequency of administration of oral iron supplementation could be of clinical importance because it may reduce the incidence of gastrointestinal side effects.**

Iron deficiency is the other main factor involved in ESA hyporesponsiveness, and iron therapy is indeed a cornerstone of treatment of CKD anemia [2–4]. Besides inhibiting hepcidin production, HIF-PHIs promote transcription of genes involved in iron metabolism, such as those encoding for duodenal cytochrome B, divalent metal transporter-2, transferrin, and transferrin receptor, thus increasing the intestinal uptake of iron and its delivery to the bone marrow [4, 20].

Although iron parameters and indexes of iron utilization were not primary outcomes in HIF-PHI trials, most RCTs showed greater reduction in serum hepcidin [15, 24, 28, 29, 40, 41, 51, 53, 54, 58, 60, 61] and ferritin concentration [24, 28, 50, 54, 58, 61] in patients randomized to HIF-PHIs, thus emphasizing their role in both improving iron mobilization and absorption. Moreover, HIF-PHIs significantly increased serum transferrin and total iron binding capacity, thus confirming better iron delivery to peripheral tissues [15, 24, 28, 29, 40, 41, 47, 51, 58, 61, 62]. From a clinical perspective, HIF-PHIs could reduce the need for iron supplementation thereby postponing the use of intravenous iron supplementation [63]. This may translate into a clinical benefit for NDD-CKD and peritoneal dialysis patients by preserving arm veins for subsequent vascular access for hemodialysis [64].

In patients with NDD-CKD, lower intravenous iron has been reported in the roxadustat arm as compared to placebo, with similar hemoglobin response in either iron-replete or iron-deficient patients, over a relatively short follow-up [29, 40, 41]. In the DOLOMITES study, the use of either oral or intravenous iron was lower in roxadustat versus darbepoetin, with comparable iron serum levels achieved [42]. In trials comparing molidustat versus darbepoetin, the amount of intravenous or oral iron resulted lower with molidustat [47, 48]. Finally, trials evaluating daprodustat, vadadustat and enarodustat in NDD-CKD did not show any difference in iron utilization compared to ESAs [28, 61, 62]. In DD-CKD, four trials with roxadustat reported lower intravenous iron need [24, 43, 50, 51]. Conversely, other HIF-PHIs seem to have less impact on iron requirements in terms of prescription or dose [7, 63].

### Kidney transplantation

#### Opinion of the group

**Evidence on the use of HIF-PHIs in kidney graft recipients remains limited and no trials are available in this population although they might be of particular interest. On the other hand, the potential increase in malignancy risk suggested for HIF-PHIs is of particular concern in this setting, where post-transplant neoplasia represents a key issue in patient morbidity and mortality. Finally, the pharmacokinetic interaction with immunosuppressive drugs is still largely unclear and needs specific evaluation.**

A substantial number of kidney transplant recipients develop anemia at various time points after transplantation, with a prevalence of ∼50% during the first six months that decreases to 23–35% in later stages [65]. Multi-factorial risk factors of post-transplant anemia include impaired graft function, iron deficiency, female gender, use of angiotensin-converting enzyme inhibitors, antibiotics, antiviral and immunosuppressive treatment and inflammation [65]. Kidney transplant recipients might present high levels of inflammation thus resulting hyporesponsive to ESAs. In addition, HIF-PHIs might modulate immune response. Indeed, there is extensive literature in the field of cancer immunology suggesting that persistent HIF activation, as a response to hypoxia in the tumor microenvironment, represents a key pathogenic factor in cancer-induced immunosuppression through an increase in cancer-specific regulatory T and B cell number and activity [66].

## Conclusions

After almost 30 years of ESA and iron use, a new therapeutic strategy has emerged for the treatment of CKD-related anemia. Benefits and drawbacks of anemia drugs are reported in Table 3. We believe that these new drugs are effective in correcting and maintaining hemoglobin levels, and are a valid alternative to ESAs, especially in NDD-CKD patients, since they very likely improve iron absorption and utilization, while in inflamed DD-CKD patients they are likely more effective in the presence of inflammation. Based on the results of several meta-analyses (not available at the time of the KDIGO conference), our opinion is that HIF-PHIs have similar safety profiles as conventional ESAs. This interpretation differs from that of KDIGO participants reporting that “….concerns surrounding cardiovascular and thrombotic risks persist.”. Longer-term safety data and/or post-marketing surveillance registries would certainly help in correctly disclosing the safety signals of HIF-PHIs. However, in everyday clinical practice we must follow EMA marketing authorization. Only Roxadustat is approved for non-dialysis and dialysis patients. In contrast, Vadadustat and Daprodustat, which are approved for dialysis patients alone, are not commercialized, thus heavily affecting the possibility to increase our experience in using these drug in everyday clinical practice.

**Table 3:** Potential advantages and disadvantages of anemia drugs (modified from [5])

Agents	Potential advantages	Potential disadvantages
HIF-PHIs	Oral dosing more convenient for NDD-CKD patients, PD, Home HD May facilitate anemia treatment in patients with NDD-CKD^a^ May improve utilization of iron for erythropoiesis, particularly useful for oral iron in NDD-CKD, PD May be more effective in chronic inflammatory states (CRP > 5 mg/l)^a^ (although we need more data) Stability at room temperature (no refrigerator need)	Difficult to monitor adherence^a^ Potential polypharmacy and drug–drug interactions^a^ - Avoiding the use too close to chelating drugs, including phosphate Less clinical experience^a^ Potential risk of enhancing tumor growth^a^ Potential risk of worsening retinopathy^a^ - Caution in patients with hyperthyroidism - Caution in patients with severe cardiovascular calcifications Potential risk of cyst growth in ADPKD^a^
ESAs	Adherence can be monitored with in-clinic administration in DD-CKD patients^a^ Extensive clinical experience^a^	Treatment requires self-injection or the help of relatives or of nurses Resistance in chronic inflammatory states^a^ Risk of enhancing tumor growth^a^ Antibody-mediated pure red cell aplasia (rare)^a^
Iron compounds	No serious adverse effects of oral iron^a^ Reduced ESA doses Reduced cardiovascular mortality in patients with concomitant heart failure Reduced cardiovascular events in patients receiving hemodialysis	If taken orally, risk of poor gastrointestinal tolerance and non-adherence to therapy^a^ If administered IV in NDD-CKD and PD risk of damaging veins of the arms eventually necessary for a fistula If administered IV, potential risk of allergic/anaphylactic reaction (rare) increasing oxidative stress or hemosiderosis (rare)^a^

*HIF-PHI* hypoxia-inducible factor–prolyl hydroxylase inhibitor, *CKD* chronic kidney disease, *NDD-CKD* non–dialysis-dependent CKD, *DD-CKD* dialysis-dependent CKD, *PD* peritoneal dialysis, *HD* hemodialysis, *CRP* C-reactive protein, *ADPKD* autosomal dominant polycystic kidney disease, *ESA* erythropoiesis-stimulating agent, *IV* intravenous

^a^Advantages and disadvantages shared with KDIGO conference conclusions [5]

The full expression of the quasi-physiological mechanism of HIFs -complete anemia correction- could not be tested in the RCTs because the hemoglobin target was mandated by the results of ESA trials and dependent guidelines. Regrettably, it is highly unlikely that these trials will be carried out and therefore pragmatically the current targets must also be applied to HIF-PHIs.

## Data Availability

Data sharing is not applicable to this article as no datasets were generated or analyzed for the current study.
